# Development of a HiBiT-tagged reporter Akabane virus and its application in antiviral drug screening

**DOI:** 10.3389/fcimb.2025.1760466

**Published:** 2026-01-14

**Authors:** Tianai Zhang, Zhongyin Liu, Yao Zhao, Jiamin Qin, Shucheng Zong, Wenke Ruan, Di Wang, Dongjie Chen, Shengkui Xu

**Affiliations:** 1 Beijing Key Laboratory of Traditional Chinese Veterinary Medicine, College of Animal Science and Technology, Beijing University of Agriculture, Beijing, China; 2 Key Laboratory of Veterinary Biological Products and Chemical Drugs, Ministry of Agriculture and Rural Affairs, Engineering and Technology Research Centre for Beijing Veterinary Peptide Vaccine Design and Preparation, Zhongmu Institutes of China Animal Husbandry Industry Co. Ltd, Beijing, China; 3 School of Agroforestry and Medicine, The Open University of China, Beijing, China; 4 Institute of Animal Inspection and Quarantine, Chinese Academy of Quality and Inspection & Testing, Beijing, China

**Keywords:** AKAV, drug screening, HiBiT, luciferase activity, reporter virus

## Abstract

Akabane virus (AKAV), an arthropod-borne pathogen causing severe reproductive failure in ruminants, poses a major threat to livestock industry. Given the lack of vaccines and antiviral drugs against AKAV, the development of rapid antiviral screening tools is urgently needed. To establish a quantifiable platform for AKAV research, we constructed a reporter virus (rAKAV-L-HiBiT) by inserting the 11-amino-acid HiBiT subunit of NanoLuc luciferase into the C-terminus of the L protein. The recombinant virus was characterized for growth kinetics, genetic stability, and luciferase activity. rAKAV-L-HiBiT exhibited a similar replication profile to wild-type AKAV and maintained stable luciferase activity over 10 passages *in vitro*. Crucially, luciferase activity of rAKAV-L-HiBiT showed a strong positive correlation with viral loads (r > 0.99), validating its utility as a convenient and rapid platform for AKAV quantification. Subsequently, we applied this platform to neutralization and antiviral compound screening assays and Remdesivir was identified as a potent inhibitor of AKAV. Taken together, our study presents rAKAV-L-HiBiT as a reliable tool for rapid antiviral drug discovery and neutralization testing.

## 1 Introduction

Akabane virus (AKAV), a member of the *Orthobunyavirus* genus in *Peribunyaviridae* family, is an arthropod-borne virus primarily transmitted by Culicoides midges (Fares and Brennan, 2022; de Souza et al., 2024). AKAV primarily causes reproductive disorders in ruminants (cattle, sheep, and goats), with clinical manifestations including abortions, stillbirths, and congenital anomalies (Kirkland, 2002; Kirkland, 2015). In China, AKAV outbreaks have been documented in multiple provinces and no licensed vaccines or therapeutics are currently available (Chen et al., 2021; Sun et al., 2024; Zhang et al., 2025). Thus, the development of vaccines and antiviral drugs remains a critical unmet need. AKAV genome comprises three single-stranded, negative-sense RNA segments: large (L), medium (M), and small (S) (Fuller et al., 1983). The three segments encodes six proteins, including RNA-dependent RNA polymerase (L protein), glycoproteins (Gn and Gc), non-structural protein (NSm), nucleocapsid protein (N), and NSs (Ishihara et al., 2016; Na et al., 2025).

The absence of efficient and rapid tools for quantifying viral titers has hindered the discovery of anti-AKAV therapeutics. Engineered reporter viruses expressing detectable markers (e.g., fluorescent proteins, luciferases) enable real-time quantitative monitoring of viral propagation, a promising alternative (Cherkashchenko et al., 2023; Nie et al., 2024). Common reporter tags include enhanced green fluorescent protein (eGFP), red fluorescent protein (RFP), and firefly luciferase. However, these tags have large molecular weights (≥23 kDa), which may disrupt viral replication (Zhang et al., 2020). The NanoLuc luciferase (NLuc) can produce luminescence with higher specificity than that of firefly or renilla luciferases (Hall et al., 2012). Moreover, the NLuc reporter can be split into a large subunit (LgBiT) and a smaller 11-amino-acid segment (HiBiT). In addition, its luminescence can be restored upon the binding of LgBiT to HiBiT. Thus, HiBiT can be introduced as a reporter tag fused with a viral protein to develop reporter viruses (Sasaki et al., 2018; Murakami et al., 2020; Zhang et al., 2023). HiBiT offers three key advantages: minimal size (1.6 kDa), high sensitivity, and high-throughput rapid screening (Nagashima et al., 2023).

In this study, we inserted HiBiT into the C-terminus of the L protein to generate rAKAV-L-HiBiT, and evaluated its performance as a screening platform. This reporter virus serves as a robust tool to accelerate antiviral drugs discovery.

## 2 Materials and methods

### 2.1 Virus strain and cells

The AKAV isolate AKAV-TJ2016 (GenBank accession numbers: MT761689.1, MT761688.1, MT755621.1), BHK-21, Vero, and BSR-T7/5 cells were all stored in our laboratory.

### 2.2 Reagents and antibodies

Alexa Fluor 568- and 488-conjugated antibodies (A-11001、A-11011), DMEM (12100046), and Lipofectamine™ 3000 transfection reagent (L3000075) were purchased from Thermo Fisher Scientific. HRP-labeled goat anti-rabbit IgG (ZB-2301), goat anti-mouse IgG (ZB-2305), and β-actin antibody (TA-09) were obtained from Zhongshan Golden Bridge Biotechnology (Beijing, China). The Nano-Glo^®^ HiBiT Lytic Detection System and Nano-Glo^®^ HiBiT Blotting System kits (N3030、N2410) were purchased from Promega. The ClonExpress^®^ II One Step Cloning Kit (C112-01) were purchased from Vazyme Biotech Co., Ltd. The FastKing cDNA First Strand Synthesis Kit (KR116-01) was purchased from Tiangen Biotech (Beijing, China). Favipiravir (T-705, HY-14768), Ribavirin (ICN-1229, HY-B0434), and Remdesivir (GS-5734, HY-104077) were purchased from MedChemExpress LLC.

### 2.3 Rescue of recombinant viruses

Overlapping PCR was used to clone the *HiBiT* gene to the C terminus of L, M, and S segments. All the primers were listed in **Table 1**. BHK-21 cells were transfected with the plasmids using Lipofectamine™ 3000 according to manufacturer’s instructions. Specifically, two sterile 1.5 mL centrifuge tubes were prepared, each containing 125 μL Opti-MEM. To one tube, 5 μL Lipofectamine 3000 was added, while the other tube received 2.5 μg plasmid DNA and 5 μL P3000™ Enhancer. Both tubes were thoroughly mixed, combined, and incubated at RT for 15 min. The resulting mixture was added dropwise to the pre-seeded cell culture plates. After 12 h, the medium was replaced with DMEM supplemented with 2% FBS, and the cells were incubated for an additional 72 h.

**Table 1:** Primers used in this study.

Primer name	Primer sequence	Application
HiBiT-S F	TGGCGGCTGTTCAAGAAGATTAGCTAATCTTAACTGATTCTCCAGTTTTCTTT	AKAV- S-HiBiT construction
HiBiT-S R	TTCTTGAACAGCCGCCAGCCGCTCACGATCTGGATACCAAATTGAGCCA	
HiBiT-M F	TGGCGGCTGTTCAAGAAGATTAGCTAATCAAATTAAAATAGACATAATGGGAGG	AKAV- M-HiBiT Construction
HiBiT-M R	TTCTTGAACAGCCGCCAGCCGCTCACTTTCAGCTTATTTTCTAGTTTGTACACTTT	
HiBiT-L F	TGGCGGCTGTTCAAGAAGATTAGCTAAATGAGTTGATAGAGATCAGTACGATTT	AKAV- L-HiBiT Construction
HiBiT-L R	TTCTTGAACAGCCGCCAGCCGCTCACAAAATCAAATTTTGATCGACCACC	
HiBiT-det-F	CAGAATTGATGGAGTGTATCC	AKAV-L-HiBiT Identification
HiBiT-det-R	GAGATCTCGGTCGACCGAATCTAAGTAGCCCGAGATGCGATG	

### 2.4 IFA

BHK-21 cells were fixed with 200 μL pre-cooled absolute ethanol at RT for 30 min followed by three washes with PBS (5 min each). Primary antibody was added and incubated at 37 °C for 60 min; after three PBS washes, secondary antibody was added and incubated at 37 °C for 60 min followed three PBS washes. The cells were observed under an inverted fluorescence microscope.

### 2.5 Luciferase activity assay

Viral stocks or AKAV-infected cells were collected, aliquoted, and added to 96-well plates (50 μL/well). The Nano-Glo^®^ HiBiT Lytic Reagent was prepared according to the manufacturer’s instructions and equilibrated to RT. Then, 50 μL reagent was added to each well containing the virus. The plate was shaken (300–600 rpm) for 10 minutes. After incubation, luminescence was measured using a multifunctional plate reader equipped with a luminescence module.

### 2.6 Growth curve of rescued virus

BHK-21 cells in 24-well plates were infected with rAKAV-L-HiBiT at 0.1 or 5 MOI. The whole samples were collected at 12, 24, 36, 48, 60, and 72 hpi, respectively. After repeated freeze-thaw cycles, the supernatants were harvested and the virus titers were measured by TCID_50_ according to the Reed-Muench method. The titer of each sample was determined at least three replicates. Meanwhile, 50 μL of each viral samples were subjected to luciferase activity assay.

### 2.7 Western blotting

BHK-21 cells were washed twice with PBS and lysed in cell lysis buffer containing protease and phosphatase inhibitors. The lysates were centrifuged, and the supernatants were mixed with loading buffer and denatured in boiled water. Proteins were separated by SDS-PAGE and transferred onto nitrocellulose membranes. The membranes were blocked with 2% BSA and then incubated with primary antibody at 4 °C overnight, followed by incubation with horseradish peroxidase (HRP)-conjugated secondary antibody at RT for 1 h. The membranes were detected using ECL substrate, and band intensities were quantified using Image J software. HiBiT-tagged proteins on nitrocellulose membranes were detected via Western blot using the Nano-Glo^®^ HiBiT Blotting System. The membranes were incubated with TBST for 4 h, followed by Nano-Glo^®^ HiBiT Blotting Reagent and overnight incubation at 4 °C. After adding the substrate, membranes were incubated at RT for 5 min and imaged with a chemiluminescence imager.

### 2.8 Serum neutralization assay

For the AKAV neutralization assay, hyperimmune serum (prepared and stored in our lab) was first two-fold serially diluted in DMEM (3 replicates per dilution). Concurrently, 50 μL of AKAV stock (100 TCID_50_/mL) was mixed with 50 μL of each diluted serum, and the mixtures were incubated for 1 h. Subsequently, each mixture was added to BHK-21 cells, followed by an additional 72 h in an incubator. Finally, the whole cells were subjected to IFA, and their luciferase activity was measured using a multifunctional microplate reader.

### 2.9 Antiviral drugs screening

BHK-21 cells seeded in 48-well plates were inoculated with rAKAV-L-HiBiT at an MOI of 0.1. Following 1 h of adsorption, the inoculum was removed and replaced with DMEM with 2% FBS and containing Favipiravir, Remdesivir, or Ribavirin at concentrations of 10 μM, 50 μM, or 100 μM, respectively. After 24 h of culture, cells were subjected to IFA to monitor the propagation of AKAV. Meanwhile, the supernatants were subjected to luciferase activity assay.

### 2.10 Statistical analysis

Statistical analysis was performed using GraphPad Prism software (version 10.0). Statistical differences were assessed using one-way ANOVA, two-way ANOVA, or unpaired t-tests. Significance is indicated by asterisks: *p < 0.05; **p < 0.01; ***p < 0.001; ****p < 0.0001.

### 2.11 Acknowledgments

This work was supported by the National Key R&D Program of China (2022YFD1802000), Youth Program of Beijing Natural Science Foundation (6254044), and Youth Talent Cultivation Project of Beijing Association for Science and Technology at Beijing University of Agriculture (BUA-QNTJ-2024005).

## 3 Results

### 3.1 Construction and rescue of HiBiT-tagged AKAV

**Figure 1:**
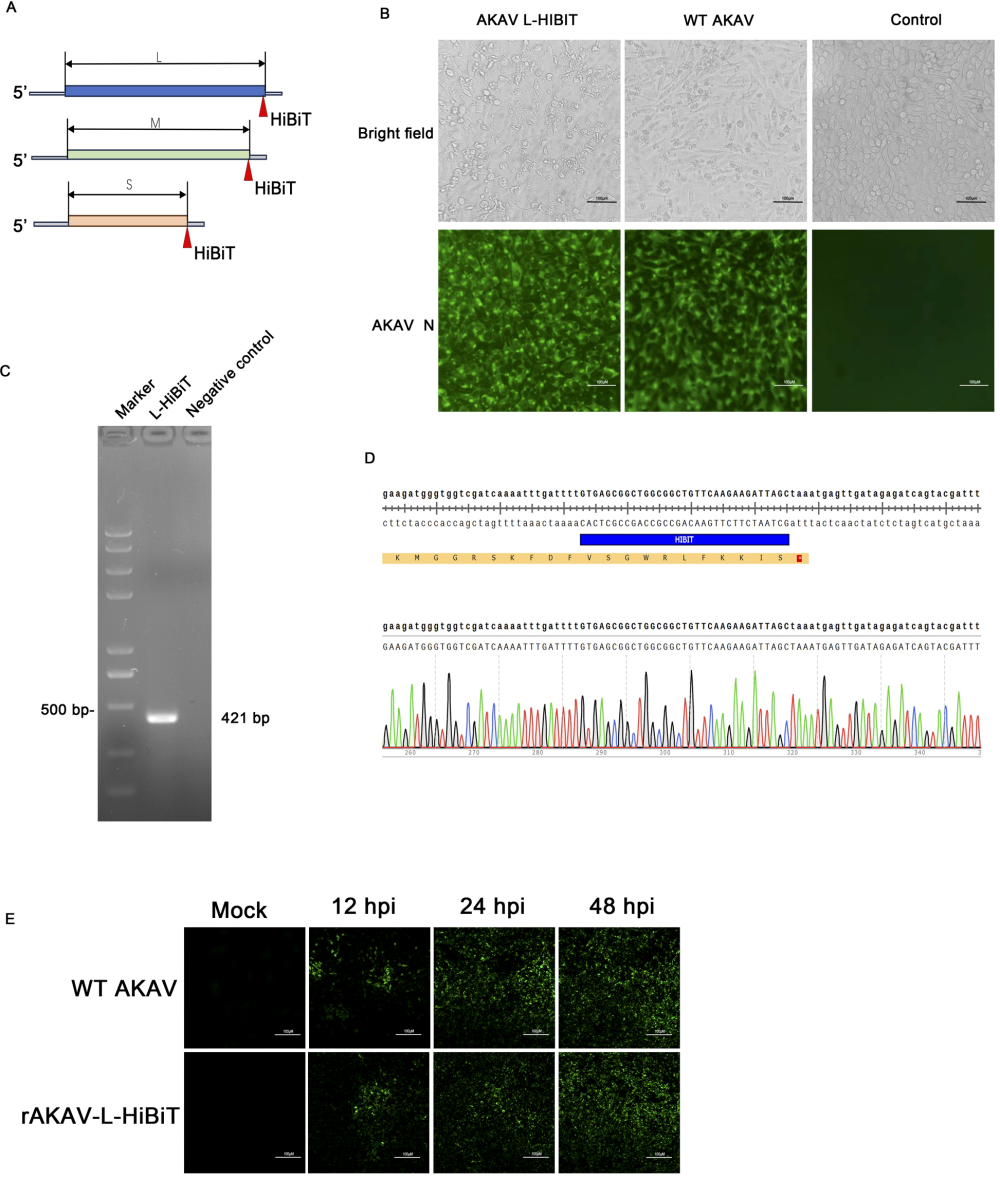
Construction and characterization of HiBiT-tagged AKAV. **(A)** Schematic diagram of the strategy for constructing recombinant AKAV expressing the HiBiT tag. The *HiBiT* was inserted in-frame at the C-terminus of the *L*, *Gc*, or *N* genes respectively. **(B)** BSR-T7/5 cells were transfected with the three plasmids (each containing only one HiBiT-tagged segment) to rescue the recombinant virus. Bright-field microscopy and IFA showed the CPE and viral N protein expression. Scale bar, 100 µm. **(C, D)** The viral RNA was extracted and subjected to RT-PCR and Sanger sequencing to identify the *HiBiT* sequence. M, DNA marker; NC, negative control. **(E)** Viral replication in Vero cells was monitored by IFA at 12, 24, and 48 hpi. Mock-infected cells served as controls.

The AKAV genome comprises L, M, and S segments, all of which have been cloned into the plasmid TVT7R, respectively (Chen et al., 2021). We successively inserted the 33-nucleotide *HiBiT* at the C-terminus of the *L*, *Gc*, and *N* genes to generate three recombinant plasmids: TVT7R-L-HiBiT, TVT7R-M-HiBiT, and TVT7R-S-HiBiT (**Figure 1A**). Then, the three plasmids, only one of which carries the *HiBiT* tag gene, were co-transfected into BSR-T7/5 cells based on the three-plasmid infectious clone system (Chen et al., 2021). At 36 hours post-transfection, the cells were harvested and passaged three times. CPE and the N signals were observed only in cells co-transfected with the TVT7R-L-HiBiT, TVT7R-M, and TVT7R-S plasmids (**Figure 1B**). Then, the presence of *HiBiT* tags was confirmed by RT-PCR and subsequent Sanger sequencing (**Figures 1C, D**). Additionally, IFA was performed using an anti-N monoclonal antibody to monitor the replication efficiency of the virus in Vero cells over time (**Figure 1E**). The results indicated that rAKAV-L-HiBiT was successfully generated and could replicate efficiently *in vitro*.

### 3.2 The genetic stability and replication capacity of rAKAV-L-HiBiT

**Figure 2:**
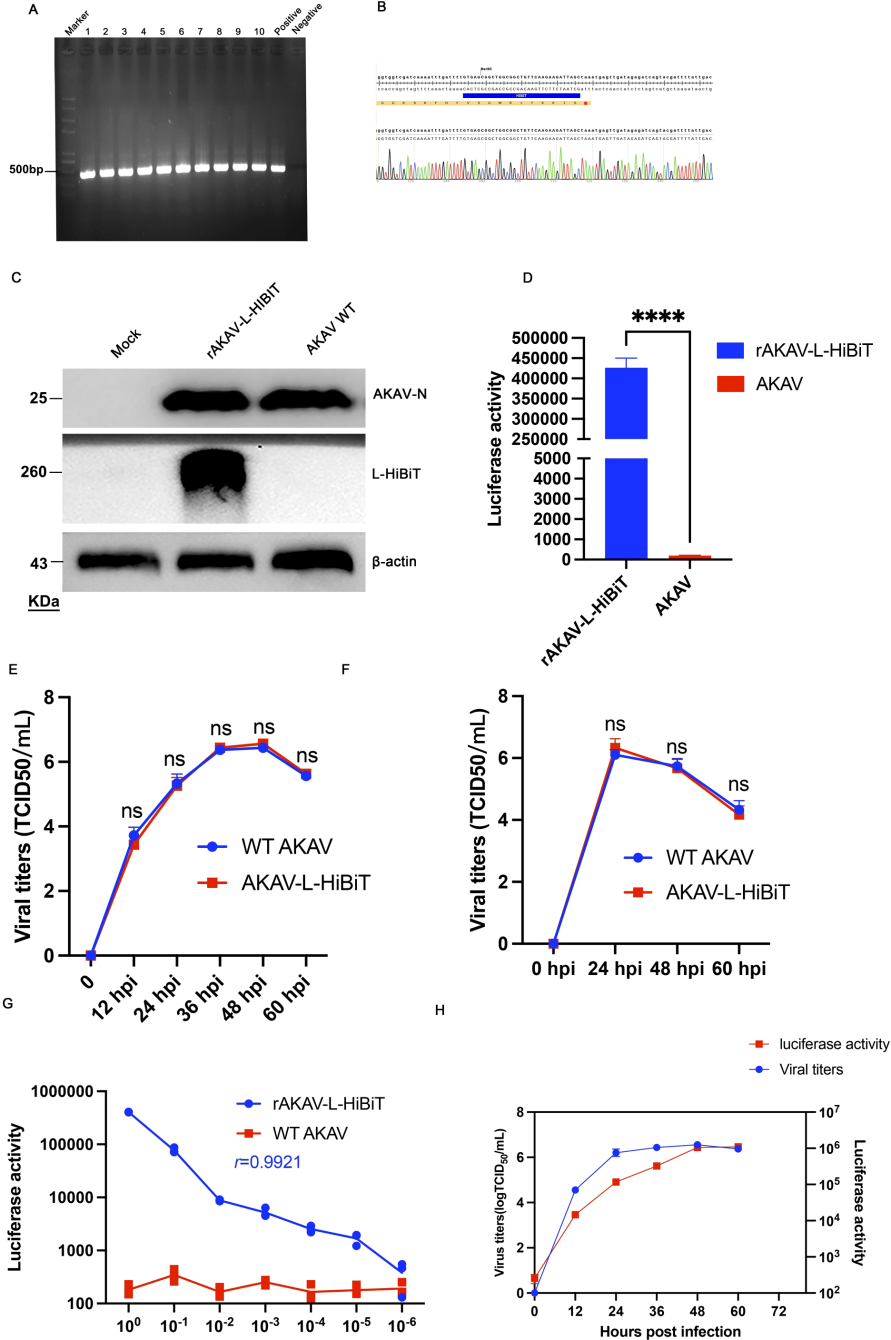
Stability, expression, and characterization of the HiBiT tag in rAKAV-L-HiBiT. **(A)** The presence of HiBiT in the rAKAV-L-HiBiT genome from passages 1 to 10 (P1–P10) was detected by RT-PCR. **(B)** The viral RNA from P10 was subjected to Sanger sequencing to identify the HiBiT sequence. **(C)** BHK-21 cells were infected with rAKAV-L-HiBiT for 24 h and the whole cell lysates were subjected to Western blotting to detect the expression of L-HiBiT recombinant protein. **(D)** The BHK-21 cells were infected with rAKAV-L-HiBiT for 24 h, then the whole cell lysis was harvested and performed luciferase activity detection by plate reader. Data are presented as mean ± SD (n=3). ****, p < 0.0001.**(E)** The rAKAV-L-HiBiT and AKAV WT stocks were serially diluted 10-fold and subjected to luciferase activity measurement. **(F)** BHK-21 cells were infected with rAKAV-L-HiBiT at an MOI of 0.1 and the cell samples were collected at the indicated time points. Viral titers and HiBiT luciferase activity were measured. All data are presented as mean ± SD (n=3).

To evaluate the stability of the *HiBiT* sequence, rAKAV-L-HiBiT was serially passaged 10 times in BHK-21 cells. RT-PCR analysis of the recombinant virus from passages 1 to 10 confirmed the presence of *HiBiT* in the viral genome (**Figure 2A**), and Sanger sequencing of the 10th passage further verified the integrity of *HiBiT* (**Figure 2B**). Next, the expression of the L-HiBiT recombinant protein was detected by Western blot and luciferase activity assay. As shown in **Figure 2C**, the L-HiBiT recombinant protein was highly expressed at 24 hpi. Additionally, the luciferase activity was approximately 2213-fold higher than that of cells infected with wild-type (WT) AKAV at 24 hpi (**Figure 2D**).

To assess the replication capacity of rAKAV-L-HiBiT, its one-step and multi-step growth curves were determined. As shown in **Figures 2E, F**, viral titers of rAKAV-L-HiBiT were comparable to those of WT AKAV. Collectively, these results indicate that the *HiBiT* fused to the C-terminus of the *L* gene is stable and does not impair viral replication. Then, we analyzed the correlations between rAKAV-L-HiBiT titers and HiBiT luciferase activity. First, the viral stocks were serially diluted 10-fold (from 1 to 10^-6^), and the luciferase activity of diluted viral stocks was analyzed. The results demonstrated a strong correlation between luciferase activity and viral titers, with a correlation coefficient (r) of 0.9921(**Figure 2G**). To monitor luciferase activity during infection, BHK-21 cells were inoculated with rAKAV-L-HiBiT, and both the viral titers and luciferase activity were detected at 12, 24, 36, 48, and 60 hpi. As shown in **Figure 2H**, viral titers reached a plateau at 36 hpi and exhibited a positive correlation with luciferase activity. Notably, the luciferase activity increased gradually from 48 to 60 hpi, whereas the viral titers remained unchanged. This discrepancy may result from the accumulation of L-HiBiT fusion proteins in non-infectious viral particles or intracellular L-HiBiT protein.

### 3.3 Applicability of rAKAV-L-HiBiT for neutralization assay

**Figure 3:**
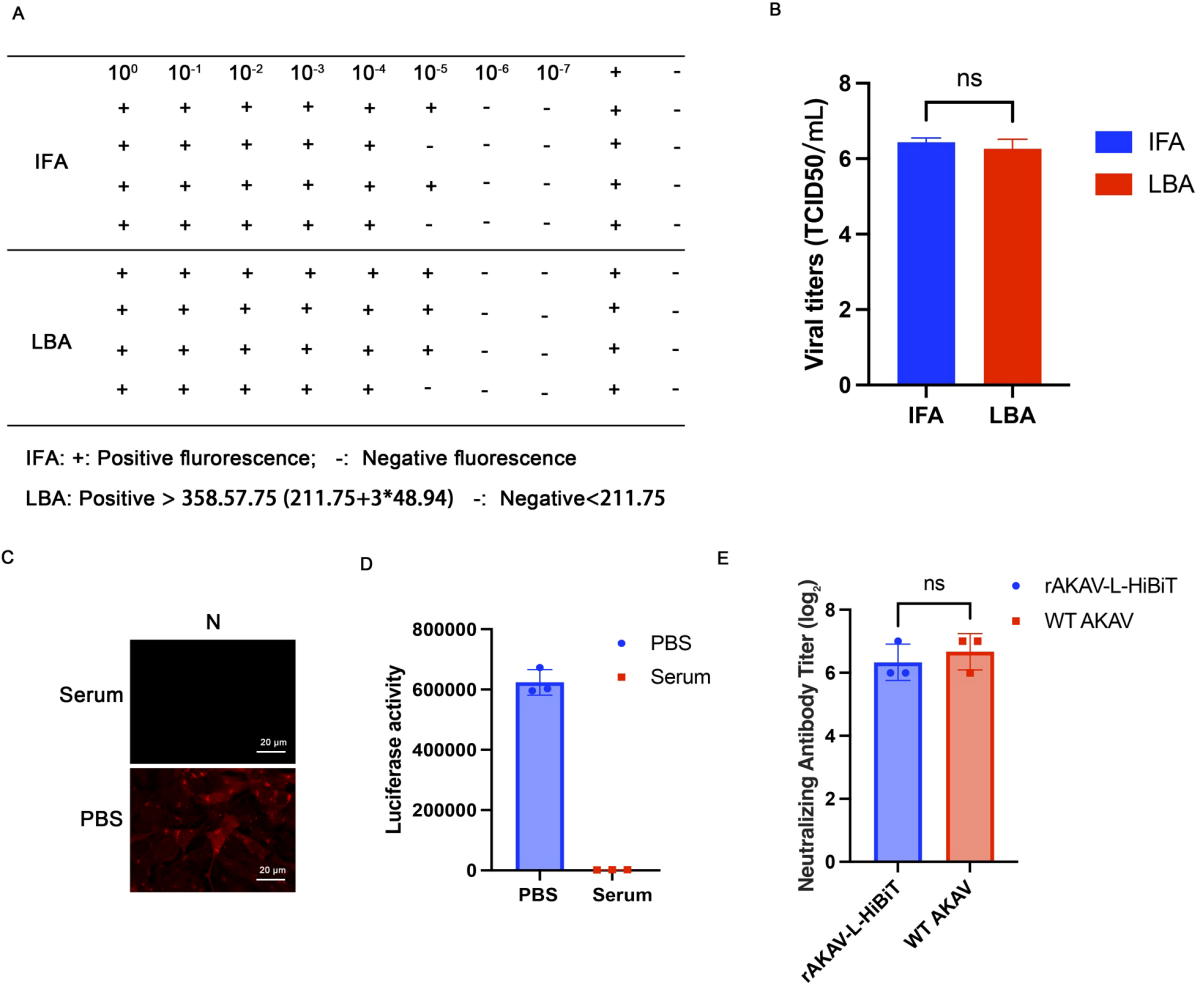
Development and Validation of a Luciferase-Based Neutralization Assay Using rAKAV-L-HiBiT. **(A)** The titers of rAKAV-L-HiBiT stock was determined via both IFA and luciferase activity assay. **(B)** Statistical analysis of the viral titers determined by luciferase activity and TCID_50_. Three independent repeats were carried out. **(C, D)** Equivalent viral stocks were pretreated with hyperimmune serum or PBS for 1 h, then inoculated onto the BHK-21 cells. 24 h later, the cells were either subjected to IFA or subjected to luciferase activity measurement (n=3). **(E)** The hyperimmune serum was serially diluted 2-fold and mixed with 100 TCID_50_ of rAKAV-L-HiBiT or AKAV WT. The neutralization rates were determined by luciferase-based assay. All data are presented as mean ± SD (n=3). ns: not significant.

While IFA remains the gold standard for quantifying neutralizing antibodies, its labor-intensive nature limits its utility. To establish a rapid alternative method, we evaluated the utility of rAKAV-L-HiBiT in neutralization assays. First, rAKAV-L-HiBiT stocks were titrated using both IFA and luciferase-based assay (LBA). As shown in **Figures 3A, B**, the viral titers showed no significant difference between the two methods.

To assess the suitability of rAKAV-L-HiBiT as a model virus, hyperimmune serum was used to neutralize both the WT AKAV and rAKAV-L-HiBiT. As shown by IFA and luciferase activity, viral replication was significantly inhibited in cells treated with the hyperimmune serum, indicating that the serum is appropriate for neutralization assays (**Figures 3C, D**). Subsequently, both rAKAV-L-HiBiT and WT AKAV were tested in hyperimmune serum neutralization assays. The results revealed no difference in neutralization rates between the two viruses, confirming that rAKAV-L-HiBiT can effectively serve as a model virus (**Figure 3E**).

### 3.4 Applicability of rAKAV-L-HiBiT for antiviral drug screening

**Figure 4:**
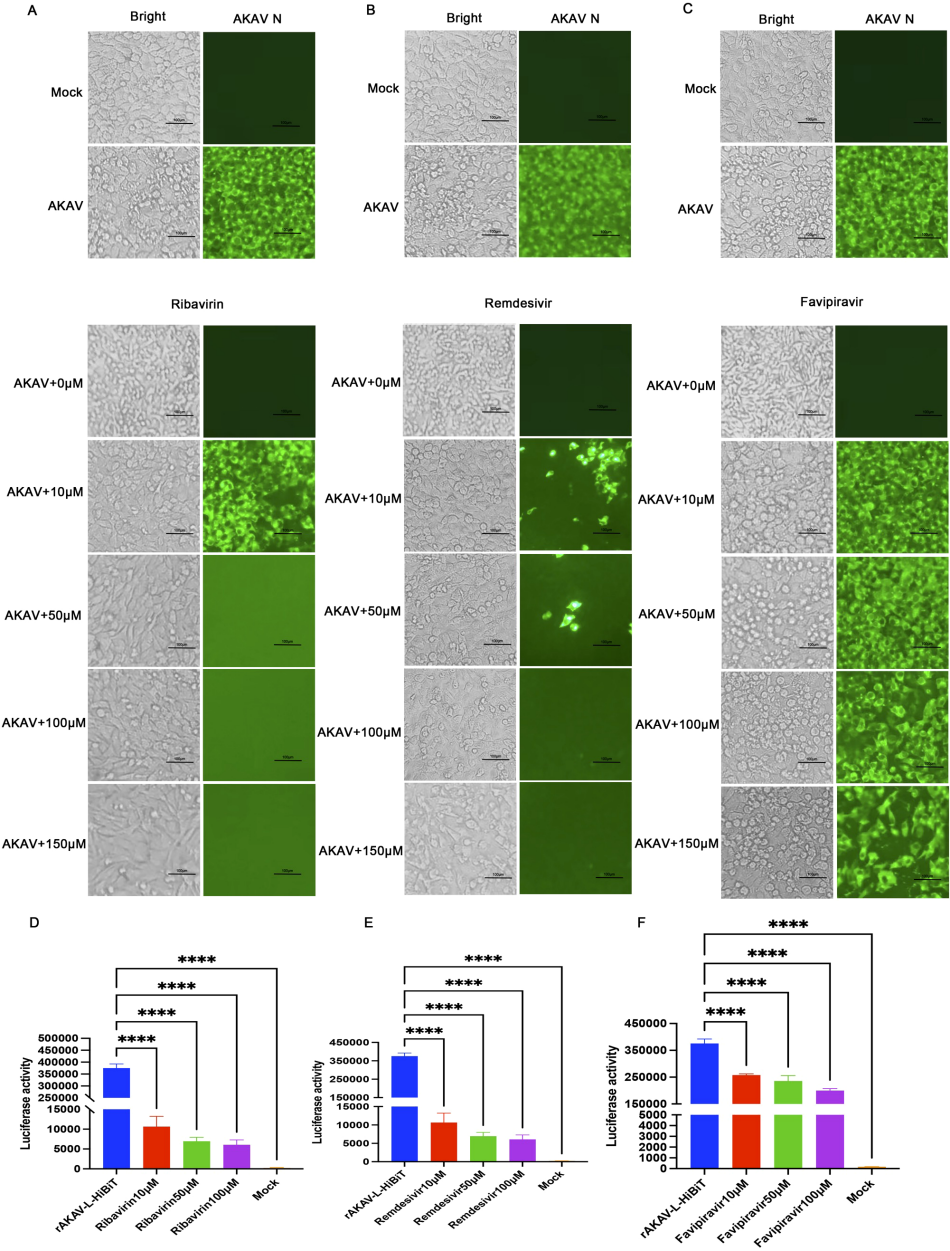
Evaluation of rAKAV-L-HiBiT as a tool for antiviral drug screening. **(A–C)** BHK-21 cells were pretreated with remdesivir, ribavirin, or favipiravir for 2 h with various concentrations. Then the cells were inoculated with rAKAV-L-HiBiT at an MOI of 0.1 with the addition of remdesivir, ribavirin, and favipiravir respectively. 24 h later, the cells were subjected to IFA with an antibody against N protein. **(D–F)** Quantitative assessment of the HiBiT luciferase activity in rAKAV-L-HiBiT-infected cells when treated with remdesivir, ribavirin, and favipiravir. All data are presented as mean ± SD (n=3).

To evaluate the suitability of rAKAV-L-HiBiT for antiviral drug screening, three antiviral compounds—ribavirin, remdesivir, and favipiravir —were tested in this study. In AKAV-infected groups, cells exhibited typical CPE at 36 hpi. In contrast, CPE was less severe in groups treated with ribavirin or remdesivir (**Figures 4A, B**). Notably, both remdesivir and ribavirin almost completely inhibited viral replication at a concentration of 150 μM, whereas favipiravir provided relatively weaker protection (**Figure 4C**).

We then detected luciferase activity of rAKAV-L-HiBiT in samples treated with the drugs. As shown in **Figure 4D**, 10 μM ribavirin reduced luciferase intensity by over 4-fold at 24 hpi, while increasing the concentration to 100 μM resulted in a more significant >30-fold reduction. Remdesivir inhibits virus replication similarly to ribavirin (**Figure 4E**), while favipiravir-treated cells showed less reduction in luciferase activity than the other two drugs (**Figure 4F**). This result provides a reliable quantitative readout but also confirms that the luciferase-tagged recombinant virus serves as a robust tool for high-throughput screening of anti-AKAV drugs.

## 4 Discussion

As a major etiological agent of diseases in ruminants, AKAV has limited available vaccines or therapeutic agents in China. In the present study, we successfully constructed a reporter virus, rAKAV-L-HiBiT, which expresses the 11-aa HiBiT tag at the C-terminus of the L protein. This recombinant virus enables the establishment of a high-throughput platform for both AKAV neutralization assays and antiviral drug screening.

Generally, inserting exogenous genes into specific sites of viral genomes or fusing them with viral non-essential genes constitutes a superior strategy for rescuing recombinant viruses. For instance, a recombinant Oropouche virus (OROV) expressing the fluorescent protein ZsGreen was constructed by replacing the NSm gene (Gunter et al., 2024). Due to the small size, HiBiT can be inserted to non-structural proteins or structural proteins without affecting viral replication. To enable quantification of the viral particles, we tried to express the HiBiT tag as a fusion with viral structural proteins (i.e., N, Gc, and L proteins). However, only the recombinant virus rAKAV-L-HiBiT was successfully rescued, indicating that inserting of the HiBiT tag at the C-termini of the N and Gc proteins adversely affects viral replication. Tagging the N or Gc protein may perturb viral packaging, as even minor insertions can disrupt molecular protein–protein interactions and undermine the basis of viral replication. We did not include flexible linkers between viral proteins and HiBiT in this study, given their potential to augment the exogenous amino acid segment length, induce immunogenicity, impair protein stability, or interfere with viral packaging. As the viral RdRp, the L protein is critical for viral replication and may tolerate insertion of small tags at its C-terminus without compromising enzymatic activity (Meier et al., 2023). We also showed that the insertion of the HiBiT tag did not significantly impair viral fitness, as demonstrated by its growth kinetics. Meanwhile, the HiBiT tag remained stable during viral replication, and so did its luciferase activity.

In our study, AKAV virions were not purified by sucrose density gradient centrifugation; thus, the detected L-HiBiT recombinant protein represents the sum of that in virions and supernatants. Consequently, luciferase activity cannot specifically reflect the L-HiBiT protein in virions. The correlation between virus titers and luciferase activity is critical for a reporter virus. Compared with GFP- or RFP-tagged reporter viruses, the titers of these fluorescently labeled viruses can only be calculated by counting fluorescent foci in specific fields of view under a fluorescence microscope (Tatineni et al., 2011; Hu et al., 2018; Wieczorek et al., 2020). This method is labor-intensive, low-throughput, and prone to bias in viral titer measurement (Wang et al., 2023). Moreover, the large tag fused with viral proteins may affects viral replication. For the HiBiT-tagged virus, the tag’s small size and high sensitivity enable flexible insertion sites and sensitive detection. By leveraging this reporter virus, researchers can now conduct high-throughput screens for neutralizing antibodies or antiviral compounds efficiently (Cho et al., 2024).

For neutralization assays, this reporter virus enables rapid quantification of antibody-mediated inhibition of viral entry or replication, eliminating the need for traditional plaque assays or IFA. Notably, this substantially accelerates the characterization of immune responses. For antiviral drug screening, the rAKAV-L-HiBiT system allows direct measurement of viral replication inhibition by candidate compounds. To validate this, we evaluated three clinically relevant antiviral agents (remdesivir, ribavirin, and favipiravir) using this system. Our results showed that at the same concentration, remdesivir exhibited the strongest inhibitory activity against AKAV, followed by ribavirin, while favipiravir showed the weakest suppression. These findings not only confirm the reliability of the rAKAV-L-HiBiT system for antiviral drug screening but also provide experimental evidence to prioritize anti-AKAV compound development.

Despite these promising features, the potential limitations of the reporter virus should not be neglected. While the HiBiT tag did not impair viral replication *in vitro*, its long-term stability *in vivo*—such as in animal models—requires further validation. Thus, its application in measuring viral loads in animal challenge models remains undetermined, though a previous study demonstrated that HiBiT-tagged viruses act as a convenient, stable tool for assessing viral propagation both *in vitro* and *in vivo (*Wang et al., 2022). Additionally, the tag’s influence on viral pathogenesis, including tropism for specific tissues or induction of host immune responses, warrants investigation. Future studies should also explore whether the HiBiT tag can be engineered into other regions of the L protein to improve this tool.

In summary, the successfully constructed rAKAV-L-HiBiT offers a sensitive, quantitative, and versatile reporter system. Its ability to support high-throughput neutralization assays and drug screening makes it an invaluable tool for accelerating AKAV therapeutic development.

## Data Availability

The datasets presented in this study can be found in online repositories. The names of the repository/repositories and accession number(s) can be found below: https://www.ncbi.nlm.nih.gov/genbank/, MT761689.1, MT761688.1, MT755621.1.
